# The study of the Bithorax-complex genes in patterning CCAP neurons reveals a temporal control of neuronal differentiation by Abd-B

**DOI:** 10.1242/bio.012872

**Published:** 2015-08-14

**Authors:** M. Moris-Sanz, A. Estacio-Gómez, E. Sánchez-Herrero, F. J. Díaz-Benjumea

**Affiliations:** Centro de Biología Molecular-Severo Ochoa (CSIC-UAM), c./Nicolás Cabrera 1, Universidad Autónoma, Madrid 28049, Spain

**Keywords:** *Drosophila*, Central nervous system, CCAP, Bursicon, HOX genes, Abdominal-B

## Abstract

During development, HOX genes play critical roles in the establishment of segmental differences. In the *Drosophila* central nervous system, these differences are manifested in the number and type of neurons generated by each neuroblast in each segment. HOX genes can act either in neuroblasts or in postmitotic cells, and either early or late in a lineage. Additionally, they can be continuously required during development or just at a specific stage. Moreover, these features are generally segment-specific. Lately, it has been shown that contrary to what happens in other tissues, where HOX genes define domains of expression, these genes are expressed in individual cells as part of the combinatorial codes involved in cell type specification. In this report we analyse the role of the Bithorax-complex genes – *Ultrabithorax*, *abdominal-A* and *Abdominal-B* – in sculpting the pattern of crustacean cardioactive peptide (CCAP)-expressing neurons. These neurons are widespread in invertebrates, express CCAP, Bursicon and MIP neuropeptides and play major roles in controlling ecdysis. There are two types of CCAP neuron: interneurons and efferent neurons. Our results indicate that Ultrabithorax and Abdominal-A are not necessary for specification of the CCAP-interneurons, but are absolutely required to prevent the death by apoptosis of the CCAP-efferent neurons. Furthermore, Abdominal-B controls by repression the temporal onset of neuropeptide expression in a subset of CCAP-efferent neurons, and a peak of ecdysone hormone at the end of larval life counteracts this repression. Thus, Bithorax complex genes control the developmental appearance of these neuropeptides both temporally and spatially.

## INTRODUCTION

Neuropeptides are small proteins, widespread in multicellular organisms, which act as chemical signals within the endocrine system. They have been most intensively studied in crustaceans and insects, where they are implicated in the control of specific behaviours and physiological functions (Nässel, 1996).

In *Drosophila melanogaster*, about 42 genes that encode precursors of neuropeptides, peptide hormones or protein hormones have been identified (Nässel and Winther, 2010). They are expressed in a stereotyped pattern of neurons and neurosecretory cells, mostly in the CNS. These are usually large cells easily identifiable by immunostaining (Park et al., 2008).

Although different segments often contain a similar set of neurons and glial cells, peptidergic neurons are segment-specific and their numbers often change during development, so that their expression is adapted to both changing physiological requirements and the development of the organism.

One paradigmatic example of a neurosecretory cell is the crustacean cardioactive peptide (CCAP)-expressing neuron. These cells produce three neuropeptides: CCAP, Bursicon (Burs) and the myoinhibitory peptides (MIPs=Allatostatin B, AstB) (reviewed in Nässel, 2002; Nässel and Winther, 2010).

CCAP is widespread in invertebrates and is involved, in addition to its cardioacceleratory action, in the control of ecdysis (Dulcis et al., 2005; Ewer, 2005; Mesce and Fahrbach, 2002; Park et al., 2003). The Burs neuropeptide, which is also found in other insects, is a tanning factor involved in the control of ecdysis. The active form of Burs is a heterodimer composed of Bursα and Bursβ. Genetic evidence shows that these CCAP/Burs-expressing neurons (hereafter referred to as CCAP neurons) play a key role in head eversion, and leg and wing expansion, at pupal ecdysis (Dewey et al., 2004; Peabody et al., 2008). CCAP neurons in the anterior-most abdominal (A) segments (A1–4) also express the myoinhibitory peptides (MIPs), which are also involved in regulating ecdysis (Kim et al., 2006). In summary, the complex set of neuropeptides expressed by these neurons confers on the latter crucial roles in the genetic networks that control the different phases of ecdysis.

**Fig. 1.**
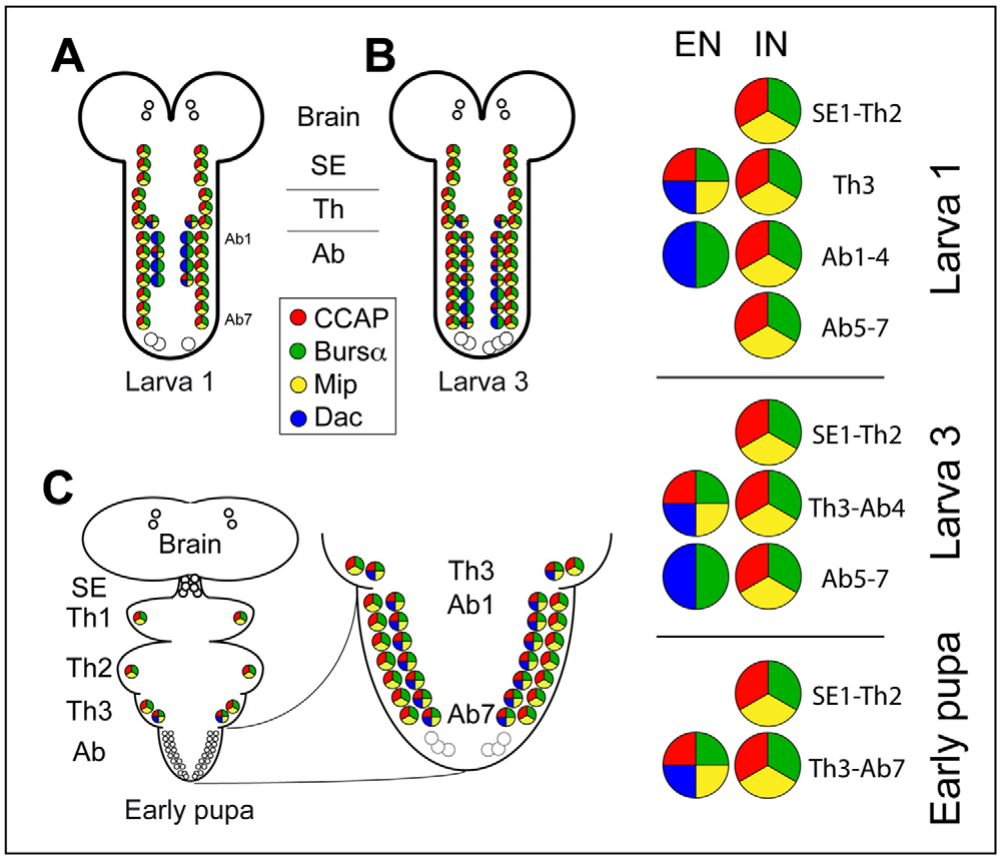
**Pattern of expression of CCAP, Bursα and MIP in the ventral nerve cord.** (A-C) Diagram of the pattern of expression of CCAP, Bursα and MIP in the CCAP neurons of first (A) and third (B) instar larvae and early pupae (C). The expression of Dac (blue) is indicated. An enlarged view is shown on the right. EN, efferent neuron; IN, interneuron; SE, subesophageal segments; Th, thoracic segments; Ab, abdominal segments. Differences in level of expression are not indicated. Due to its enormous variability, the expression of these genes in segments A8–9 is not indicated.

There are two types of CCAP neurons in the ventral nerve cord (VNC) of the fly: interneurons (CCAP-INs) and efferent neurons (CCAP-ENs). Three main features distinguish these two types of neurons: expression of the gene *dachshund* (*dac*) in the ENs, the efferent axons of the ENs, which exit the ganglion via the lateral segmental nerve, and expression of the gene *hunchback* in the INs (Moris-Sanz et al., 2014; Veverytsa and Allan, 2012). In the VNC of first instar larvae, CCAP-INs expressing both CCAP and Bursα are present in the subesophageal (SE1–3), thoracic (T1–3) and first seven abdominal segments (A1–7), one per hemisegment (Fig. 1A). At this stage, CCAP-ENs are only found in segments T3–A4. Of these cells, only the cells of the T3 segment and, generally, one of the abdominal ones, express CCAP (Fig. 1A). This pattern changes over time and, indeed, in third instar larva, all the CCAP-ENs of T3–A4 segments express both CCAP and Bursα. Later, in early pupal development, one extra CCAP-EN expressing both neuropeptides appears in each of the A5–7 hemisegments, and these last neurons have been called “late” CCAP*-*ENs (Veverytsa and Allan, 2012). During pupal development, three more ENs appear in hemisegments A8–9, the “posterior late” CCAP-ENs (Fig. 1; supplementary material Fig. S1).

MIP neuropeptides (MIP1–5) are encoded by the gene *mip* (Williamson et al., 2001). MIP expression had been reported previously by northern blots, weak in embryos and stronger in third instar larvae, pupae and adults (Williamson et al., 2001). Their expression pattern has been shown in late third instar larvae as restricted to a subset of the CCAP neurons (Kim et al., 2006). In first instar larvae we found MIP expression in all the CCAP-INs as well as in the CCAP-ENs that express CCAP at this stage, although the expression is weak (Fig. 1A). In late third instar larvae and early pupae MIP expression is stronger and follows the same pattern as CCAP (Fig. 1B-C; supplementary material Fig. S1C-D).

Although the onset of expression of these neuropeptides in CCAP neurons is quite dynamic, all these neurons are generated from the same progenitor neuroblast (Moris-Sanz et al., 2014; Veverytsa and Allan, 2012). This raises two important questions: first, how is the segmental pattern of CCAP-ENs established, and second, how is terminal differentiation of these neurons temporally tuned.

In this report we address the role of the genes of the BX-C: *Ubx*, *abd-A* and *Abd-B*, in control of the spatial and temporal patterning of CCAP-ENs. From an analysis of phenotypes in conditions where the BX-C genes are inactive or misexpressed we conclude: first, that CCAP-ENs are specified in all segments from SE1 to A7 during embryonic neurogenesis but die by apoptosis in segments SE1–T2, and this programme of cell death is rescued by the action of Ubx in segments T3–A1 and by Ubx and Abd-A in segments A2–7; thus, Ubx and Abd-A play crucial roles in the specification of these neurons. Second, Abd-B is not required for specification of these neurons, but temporally represses expression of the neuropeptides in the CCAP-ENs of segments A5–7, without affecting their expression in the CCAP-INs. Thus, although these ENs are specified, as in the other segments, during embryonic neurogenesis, the start of neuropeptide production by them is delayed until the early pupa. Thus, the BX-C genes, by various mechanisms, spatially and temporally modulate the expression of neuropeptides in the CNS.

## RESULTS

### Requirements of Ubx and Abd-A in the specification of the CCAP-ENs

Because of the segmental differences observed in the pattern of CCAP-ENs, we wondered whether HOX genes played a role in controlling the specification of CCAP-ENs. We first examined the role of Ubx and Abd-A.

**Fig. 2.**
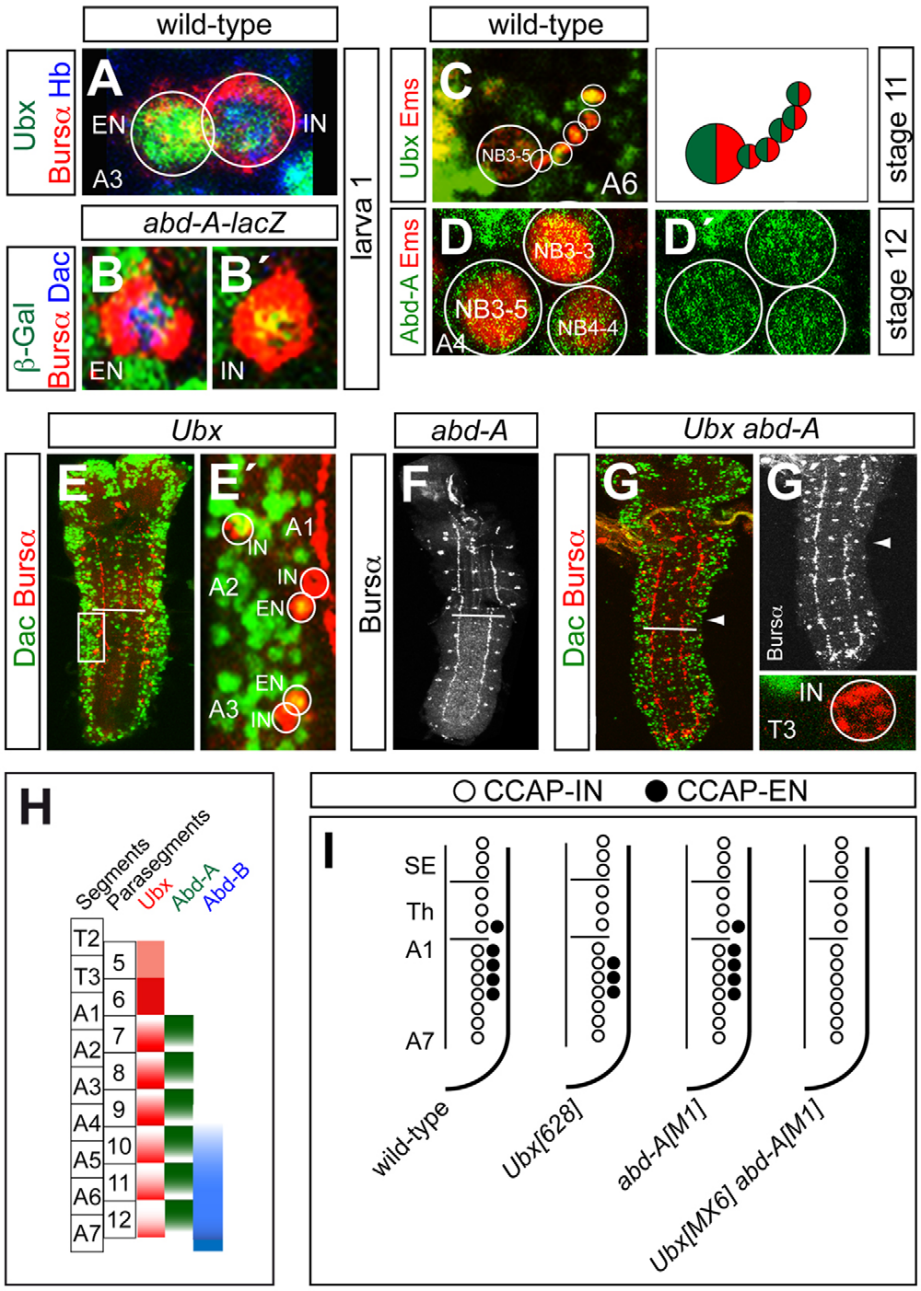
**Pattern** **of CCAP neurons in *Ubx* and *abd-A* mutants.** (A-B′) Expression in the CCAP neurons of segment A3 of (A) Ubx (green), Bursα (red) and Hb (blue); (B,B′) β-Galactosidase (green), Bursα (red) and Dac (blue) expression in *abd-A-lacZ*. Here, the CCAP-IN is identified by stronger expression of Hb (Moris-Sanz et al., 2014). (C) Expression of Ubx (green) and Ems (red) in the NB3-5 cluster of stage 11; a drawing depicting the observed pattern is shown on the right. (D-D′) Expression of Abd-A (green) and Ems (red) in the Ems-expressing NBs (3-3, 3-5 and 4-4) at stage 12. Abd-A expression is weak in all NBs. (E) Expression of Dac (green) and Bursα (red) in *Ubx^6.28^*. (E′) Magnified view of the boxed area in E. (F) Expression of Bursα in *abd-A^M1^*. (G) Expression of Dac (green) and Bursα (red) in *Ubx^MX6^ abd-A^M1^*. Bursα expression is shown separately in gray (G′). A higher magnification view of segment T3 (arrowhead) is shown at the bottom. (H) Diagram of the pattern of expression of Ubx (red), Abd-A (green) and Abd-B (blue) along the anteroposterior axis of the *Drosophila* VNC. The segmental and parasegmental units are indicated. (I) Summary of phenotypes. White bars in (E-G) indicate the boundary between thoracic and abdominal segments. T, thorax; A, abdomen.

In the CNS the expression of Ubx is weak in parasegment (PS)5, strong in PS6 and weakens progressively in more posterior segments (Beachy et al., 1985; White and Wilcox, 1985). This posterior decline of Ubx expression is caused by both Abd-A and the expression of non-coding RNAs harboured in the BX-C (*iab-4* and *iab-8*) (Bender, 2008; Struhl and White, 1985; Thomsen et al., 2010). Abd-A expression is detected from PS7 to PS12 (Karch et al., 1990; Macias et al., 1990). In each PS, Abd-A expression is stronger in the more anterior cells, where it turns off Ubx, thus resulting in complementary PS patterns (Gebelein and Mann, 2007; Karch et al., 1990) (Fig. 2H).

To study the contribution of Ubx and Abd-A to patterning of the CCAP neurons, we stained for them in first instar larvae. In these experiments, for antibody compatibility we used anti-Hunchback (Hb) to identify the ENs, as Hb is expressed more strongly in the INs (Moris-Sanz et al., 2014). We found that Ubx is expressed in both INs and ENs, strongly in A1 segments, weakly in T3 and A2–4 and barely detectably in the most posterior segments (Fig. 2A; supplementary material Fig. S2A and C). We did not detect any Abd-A expression with anti-Abd-A antibody but observed expression in both cell types in an *abd-A-lacZ* reporter line (Fig. 2B-B′; supplementary material Fig. S2B-B′), probably due to residual expression of the *lacZ* transgene.

Next, we looked for their expression in stage 11/12 embryos. CCAP neurons are generated in the Hb-temporal window of the NB3-5, which delaminates in late stage 8 of embryogenesis, and its lineage can be identified by expression of *empty spiracles* (*ems*) (Doe, 1992; Moris-Sanz et al., 2014). We observed that all the cells expressing Hb in the NB3-5 lineage, from segments T3 to A7, expressed Ubx, but that expression of Abd-A was scarcely detectible in the NB and its lineage (Fig. 2C-D′ and data not shown).

Next, we stained for Bursα in *Ubx* and *abd-A* mutants. In the null allele *Ubx^6.28^* the CCAP-ENs of T3–A1 were lost, although the CCAP-INs were not affected; on the other hand there was no change in the pattern of CCAP neurons in the null allele *abd-A^M1^* (Fig. 2E-F,I; supplementary material Table S1). In the strong double mutant allelic combination *Ubx^MX6^ abd-A^M1^* all the ENs were lost (Fig. 2G-G′,I). We obtained the same results when we stained for CCAP (supplementary material Fig. S2D-E). Together, these results suggested that Ubx is required to specify the CCAP-EN fate in segments T3–A4, and that in the absence of Ubx, Abd-A drives the specification of the ENs in segments A2−4.

These results also suggested that either Ubx represses the expression of *abd-A* in the CCAP-ENs of segments A2−4, or both genes are co-expressed in these segments but our antibody scarcely detects the low level of Abd-A expression. Repression of *abd-A* by Ubx would be surprising, as it has been reported that the pattern of Abd-A expression is not altered in *Ubx* mutants (Karch et al., 1990). However, misexpression of *Ubx* is able to repress *abd-A* expression (Castelli-Gair Hombria et al., 1994; Gebelein and Mann, 2007), which indicates that repression can occur in some contexts. On the other hand, Ubx and Abd-A are co-expressed in other neurons such as the abdominal leucokinergic neurons (ABLKs) (Estacio-Gomez et al., 2013).

To gain further insight into Abd-A expression in CCAP neurons we stained for Abd-A in *Ubx* mutants, but we failed to observe any expression in the CCAP neurons of first instar larvae (data not shown). Therefore, we stained for Abd-A in stage 9–11 embryos and found that its expression in segments A2–7 was the same as in wild-type embryos, namely weak in both NB3-5 and its progeny, although the level of expression was not the same in all neurons (data not shown). These results indicate that, in the absence of Ubx the low level of Abd-A expression detected is sufficient to specify CCAP neuronal fate.

### The roles of Ubx and Abd-A in CCAP neuron specification differ

**Fig. 3.**
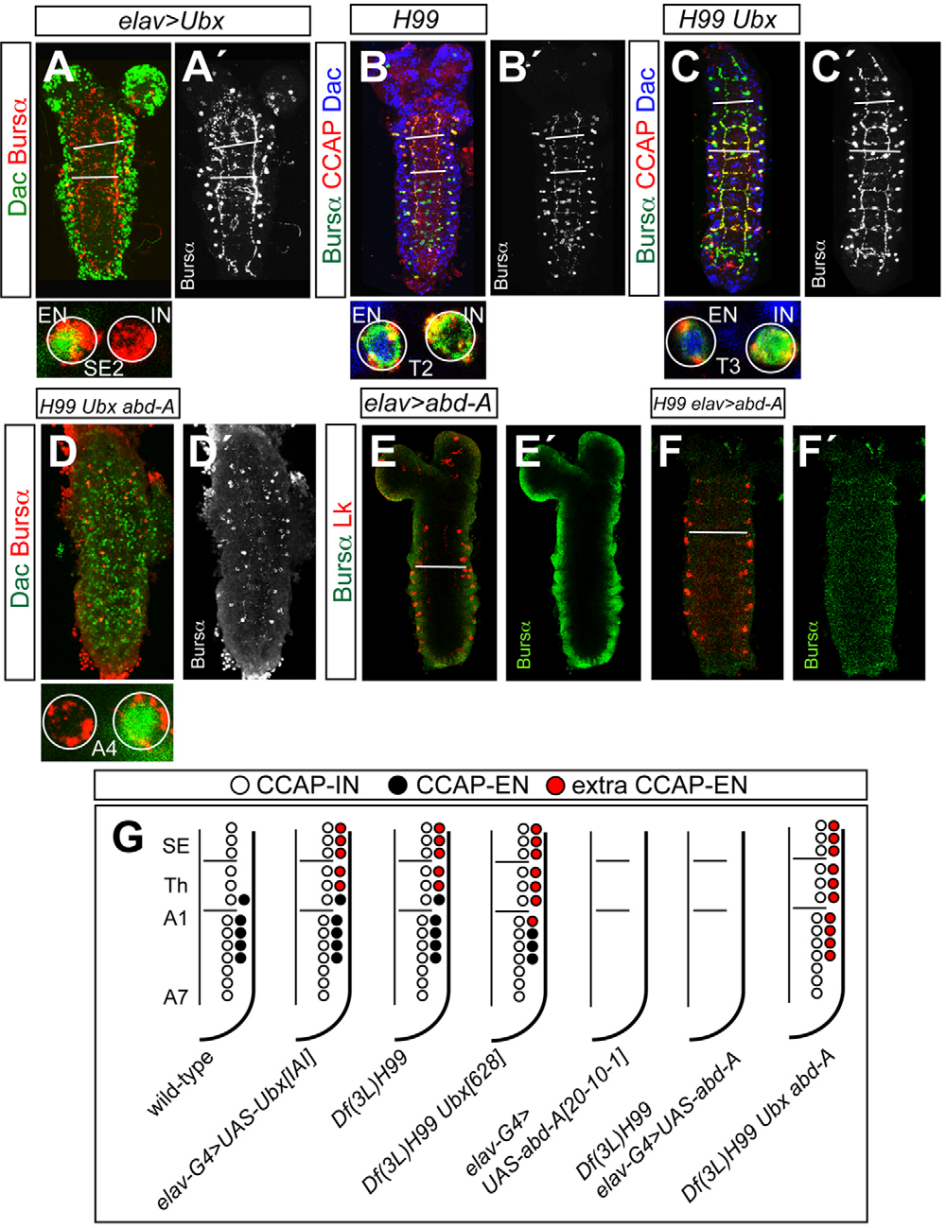
**Effects of misexpressing *Ubx* or *abd-A* on the pattern of CCAP neurons.** (A,A′) Expression of Dac (green) and Bursα (red) in *elav-Gal4 UAS-UAS-Ubx^IAI^*. (B-C′) Expression of Bursα (green), CCAP (red) and Dac (blue) in *Df(3L)H99* (B,B′) and *Df(3L)H99 Ubx^6.28^* (C,C′). (D,D′) Expression of Dac (green) and Bursα (red) in *Df(3L)H99 Ubx^6.28^ abd-A^M1^*. Closer views of segments are shown at the bottom of each figure. (E-F′) Bursα (green) and Lk (red) expression in *elav-Gal4 UAS-abd-A* (E,E′) and *Df(3L)H99 elav-Gal4 UAS-abd-A* (F,F′). Bursα expression is shown separately on the right of each figure. All samples are first instar larva. (G) Summary of phenotypes. White bars indicate boundaries between subesophagic, thoracic and abdominal segments (A-C′) or between thoracic and abdominal segments (E,F).

Next, we assessed the effect of misexpressing *Ubx* in post-mitotic cells. To that end we used *elav-Gal4* (*elav-Gal4 UAS-UAS-Ubx^IAI^*). Although it has been reported that *elav-Gal4* is also expressed in neuroblasts and therefore behaves as a pan-neural driver in late stages of neurogenesis, in earlier stages, when CCAP neurons are generates, its expression is restricted to postmitotic cells (Berger et al., 2007), thus in these experiments can be consider a postmitotic driver. We observed one extra CCAP-EN per hemisegment in segments SE1-T2, while the fate of the CCAP-INs was unaffected (Fig. 3A-A′,G). This phenotype was also observed in programmed cell death-deficient larvae (*Df(3L)H99*) (Fig. 3B-B′). These findings suggest that, in normal development, the CCAP-ENs in segments SE1–T2 die by apoptosis but can be rescued by misexpression of Ubx. In accord with this conclusion, CCAP-ENs were present in all segments from SE1 to A4 in both *Df(3L)H99 Ubx^6.28^* and *Df(3L)H99 Ubx^MX6^ abd-A^M1^* larvae (Fig. 3C-D′). Thus, preventing cell death not only rescues the CCAP-ENs of segments SE1–T2 but also those lost in segments T3–A1 in the *Ubx* mutant and in segments T3–A4 in the *Ubx abd-A* double mutant.

To see whether Ubx prevents the death of CCAP-ENs by counteracting a possible action of the anterior-most HOX genes in promoting apoptosis of the CCAP-ENs, we stained for CCAP in mutants of *labial* (*lab^1^*/*lab^4^*), *proboscipedia* (*pb^10^*), *Deformed* (*Dfd^10^*), *Sex combs reduced* (*Scr^4^*) and *Antennapedia* (*Antp^14^*/*Antp^25^*), but did not observe any phenotype (supplementary material Fig. S3A-E). Nor did we find any phenotype misexpressing these genes with *elav-Gal4* (supplementary material Fig. S3F-J). Thus, we conclude that none of these genes play any role in specification of the CCAP-ENs.

To confirm that Ubx and Abd-A play the same role in CCAP-EN specification, we misexpressed *abd-A* (*elav-Gal4 UAS-abd-A^20-10-1^*) and, surprisingly, found that all the CCAPs were lost (Fig. 3E-E′). We obtained the same result with another transgenic line in which there was a lower level of expression (*UAS-abd-A^20-1-1^*; data not shown).

These results suggest that Abd-A acts differently from Ubx. Thus, in *Ubx* mutants low levels of Abd-A rescue the CCAP-ENs, whereas higher levels cause the loss of all CCAP neurons. These results were unexpected as the number of neurons expressing the neuropeptide Leucokinin (Lk) increased in the same larvae (Fig. 3E; supplementary material Fig. S3K-L) (Estacio-Gomez et al., 2013); this suggests that the effect of Abd-A on CCAP neurons is cell-specific.

We wondered whether *abd-A* misexpression causes the death of CCAP-ENs. To test this idea we simultaneously misexpressed *abd-A* and prevented apoptosis (*Df(2R)H99 elav-Gal4 UAS-abd-A^20-10-1^*), and found that the CCAP neurons were not rescued (Fig. 3F-F′).

We consider two possible explanations for this outcome: either Abd-A represses the expression of the neuropeptide or it alters the fate of the neurons. To distinguish between these two possibilities we misexpressed *abd-A* with *CCAP-Gal4* (*CCAP-Gal4 UAS-abd-A^20-10-1^ UAS-GFP*). This driver is expressed in all CCAP neurons from second instar larva onwards. We reasoned that if Abd-A repressed the expression of the neuropeptide it would do so at any time in development, whereas a change of neuronal fate would be likely to occur only in early development. Hence, if misexpression of Abd-A during larval development were not able to repress CCAP expression this would favour the idea that Abd-A alters the fate of the CCAP, and that is indeed what happened (supplementary material Fig. S3M).

### Ubx is not required to maintain CCAP-neuronal fate

**Fig. 4.**
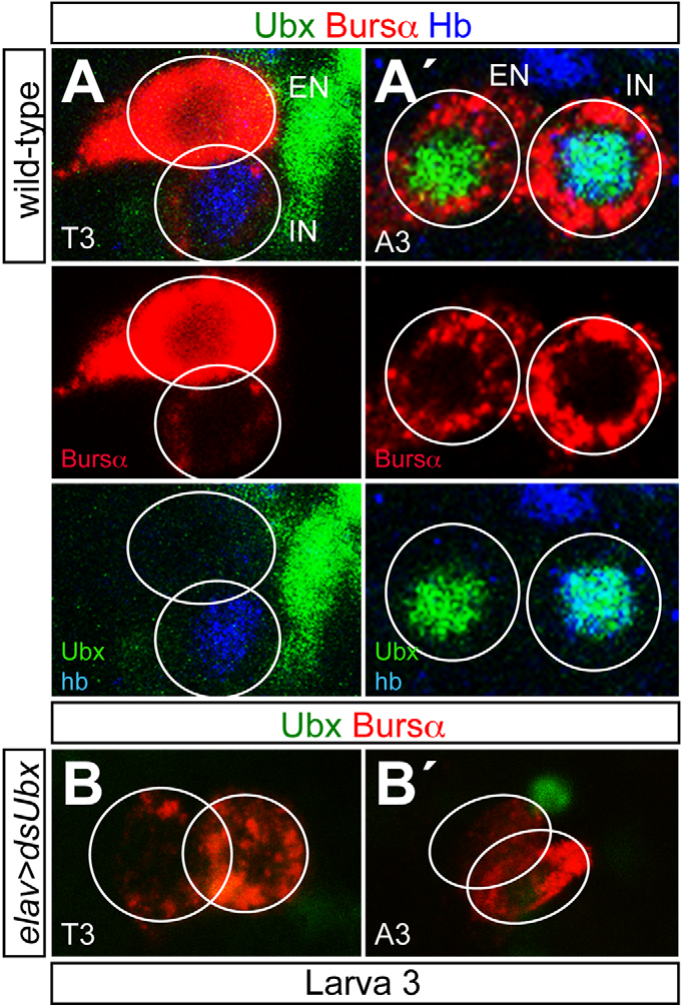
**Ubx is not required to maintain neuropeptide expression.** (A,A′) Expression of Ubx (green), Bursα (red) and Hb (blue) in the T3 (A) and A3 (A′) segments of a wild-type ganglion. (B,B′) Expression of Ubx (green) and Bursα (red) in the T3 (B) and A3 (B′) segments of a *Df(3R)109/+ elav-Gal4 UAS-dsUbx UAS-dicer2* ganglion.

A number of studies have shown that some genes involved in the initial specification of neuronal identity are also required for its maintenance in mature CNS (reviewed in Deneris and Hobert, 2014). In *Drosophila* this has been reported for a few cases (Eade et al., 2012; Estacio-Gomez et al., 2013; Hsiao et al., 2013; Jafari et al., 2012). We assessed whether maintenance of CCAP-EN identity requires permanent expression of Ubx. To this end, we stained for Ubx in third instar larvae and observed expression in the CCAPs of segments A1–4 but not in T3 (Fig. 4A). Next, we knocked down *Ubx* from first instar larva in individuals hemizygous for the BX-C (*Df(3R)109/+ elav-Gal4 UAS-dsUbx UAS-dicer2*) and did not observe any change in the pattern of Bursα expression in third instar larvae (Fig. 4B). Although we still observed low levels of Ubx expression in the CCAP neurons, this was probably due to perdurance of the protein made in the embryo, since Ubx expression is strongly inhibited in the imaginal discs of this line (D. Garaulet, personal communication). Taken together these findings support the conclusion that Ubx is not permanently required to maintain the fate of these neurons.

### Abd-B controls the onset of CCAP/Bursα expression in the efferent neurons

Although all CCAP neurons are generated during embryonic neurogenesis, neuropeptide expression in the ENs of segments A5–7 only starts in late third instar larvae/early pupae (supplementary material Fig. S1) (Veverytsa and Allan, 2012). This suggests that HOX genes could play a role, not only in the spatial distribution of these neurons, but also in the time of onset of neuropeptide expression.

In the embryonic VNC, Abd-B expression is weak in PS10 (A5) and gradually strengthens in more posterior segments (Birkholz et al., 2013; Celniker et al., 1989, 1990; de Lorenzi and Bienz, 1990) (Fig. 2H). As CCAP-ENs cannot be identified in segments A5–7 in stage 17, we directed our attention to the expression of Abd-B in CCAP-INs at different times of development and observed that Abd-B was never expressed (supplementary material Fig. S4A-C). Next, we examined its expression in the NB3-5 cluster in stages 12, 14 and 16 but did not detect any expression (supplementary material Fig. S4D-F).

**Fig. 5.**
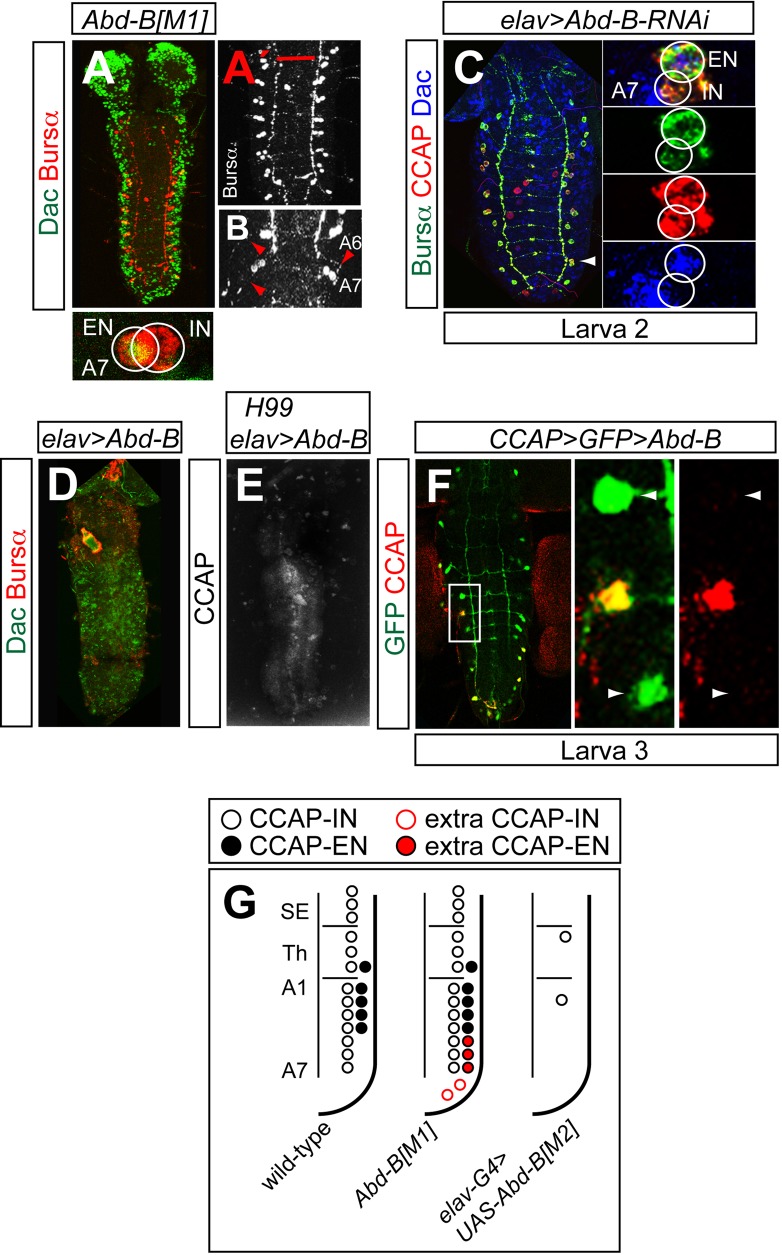
**Abd-B represses the expression of CCAP and Bursα.** (A,B) Expression of Dac (green) and Bursα (red) in *Abd-B^M1^* ganglia of larvae 1. Higher magnification views of Bursα expression in abdominal segments (A′) and in segments A6–7 (B) are shown in white. The red bar indicates the thorax/abdomen separation. Note in B the presence of two neurons per hemisegment and the efferent axons (red arrowheads). (C) Expression of Bursα (green), CCAP (red) and Dac (blue) in *elav-Gal4 UAS-Abd-B-RNAi UAS-dicer2* larvae 2. A higher magnification view of segment A7 (arrowhead) is shown on the right. (D) Expression of Dac (green) and Bursα (red) in *elav-Gal4 UAS-Abd-B^M2^*. Bursα expression is absent in both INs and ENs. (E) CCAP expression in *Df(3L)H99 elav-Gal4 UAS-Abd-B^M2^.* (F) Expression of GFP (green) and CCAP (red) in *CCAP-Gal4 UAS-GFP UAS-Abd-B^M2^* in larva 3. A higher magnification view of the boxed area is shown on the right. Arrowheads point to two CCAP neurons not expressing the neuropeptide. (G) Summary of phenotypes.

To directly test whether Abd-B has a role in the specification of CCAP/Bursα in segments A5–7, we stained for Bursα in the strong hypomorph allele *Abd-B^M1^*. This allele removes both *Abd-B* isoforms (m and r) (Casanova et al., 1986). In *Abd-B^M1^* first instar larvae we observed one EN per hemisegment in segments A5–7 and also two extra CCAP neurons in segments A8-9 (Fig. 5A-A′and G). These ENs in segments A5–7, which normally undergo terminal differentiation expressing CCAP and projecting efferent axons in pupae, possessed efferent axons in larva 1 (Fig. 5B). We obtained the same result with other strong *Abd-B* alleles (*Abd-B^Df(3R)C4^*, *Abd-B^D18^*, *Abd-B^D16^* and *Abd-B^M5^*; supplementary material Fig. S4G-I and data not shown). CCAP-ENs seem to be very sensitive to the levels of Abd-B expression because in *Abd-B^M5^*/+ first instar larvae we observed CCAP-ENs in segments A5 (supplementary material Fig. S4J).

To check whether Abd-B is permanently required to repress CCAP/Bursα expression, we knocked down *Abd-B* in first instar larvae (*elav-Gal4 UAS-Abd-B-RNAi UAS-dicer2*; although this driver is expressed in the embryo, RNAi is not effective until the larval stages) and, in second instar larvae we observed several cases in which ENs of segments A5–7 expressed neuropeptides prematurely (Fig. 5C).

When we misexpressed *Abd-B* using a pan-neuronal driver (*elav-Gal4 UAS-UAS-Abd-B^M2^*) we observed that expression of CCAP and Bursα in both INs and ENs was abolished in all segments (Fig. 5D,G and data not shown). We obtained the same phenotype when *Abd-B* was misexpressed in a genetic background preventing apoptosis (*Df(3L)H99 elav-Gal4 UAS-UAS-Abd-B^M2^*; Fig. 5E), which showed that Abd-B was not causing these cells to undergo PCD.

We also misexpressed *Abd-B* with *CCAP-Gal4* (*CCAP-Gal4 UAS-UAS-Abd-B^M2^ UAS-GFP*). This driver is expressed from second instar larva onwards. In third instar larvae we observed that CCAP expression was lost in most of these cells, which were identified by perdurance of GFP. These neurons, although they failed to express the neuropeptide, had normal morphology (Fig. 5F).

Together these results suggest that the role of Abd-B in the CCAP-EN is to delay its terminal differentiation until early pupa. Thus, if Abd-B is removed, the onset of CCAP/Bursα expression in these neurons occurs at the same time as in the other CCAP-expressing neurons, and, if it is misexpressed, CCAP/Bursα expression fails in all neurons.

### Abd-B prevents the production of Leucokinin and Corazonin neuropeptides

Next, we sought to test whether Abd-B also act on the production of other neuropeptides. In normal development, the neuropeptide Lk is expressed in one cell per hemisegment in segments A1–7; in *Abd-B^M1^* mutants, one extra expressing cell appears in A8, and when misexpressed, the expression of the neuropeptide is lost in all segments (supplementary material Fig. S5A-C) (Estacio-Gomez et al., 2013). The same effect was observed for production of the neuropeptide Corazonin (Crz). In first instar wild-type larvae, Crz is expressed in segments T2–A6; in *Abd-B^M1^*, Crz production was extended to segments A7–8, and when *Abd-B* was misexpressed it was prevented (supplementary material Fig. S5E-G). The effects of *Abd-B* misexpression were not rescued in a genetic background preventing apoptosis (*Df(3L)H99 elav-Gal4 UAS-UAS-Abd-B^M2^*; supplementary material Fig. S5D,H).

Together, these results indicate that Abd-B prevents the production of several neuropeptides. It remains to be seen whether it represses the expression of Lk and Crz, as it is the case of the CCAP and Bursα in CCAP-ENs, or alters the fate of the neurons that express them.

### Abd-B does not alter the expression of the gene *dimmed*

Following our observation that Abd-B prevents the expression of several neuropetides, we assessed whether Abd-B acts on a global factor required for the expression of neuropeptides.

The gene *dimmed/Mist1* (*dimm*) encodes a bHLH factor that is expressed in neuroendocrine cells. Genetic analysis indicated that Dimm is required in neuroendocrine cells to produce, maintain and release large stores of secretory peptides (Hewes et al., 2003). Dimm is expressed in most of the CCAP neurons, but in a *dimm* mutant (*dimm^rev7^*) the expression of CCAP was not affected (supplementary material Fig. S6A-B). We tested whether expression of Dimm was altered in *Abd-B* mutants or when *Abd-B* was misexpressed, but we did not observe any change in the pattern of Dimm expression (supplementary material Fig. S6C-D).

Since we know of no other gene that plays a global role in the differentiation of peptidergic neurons, an alternative hypothesis is that Abd-B physically interacts with the promoters of the CCAP and Burs-encoding genes. Additional experiments are needed to test this hypothesis.

### The ecdysone pathway drives CCAP-EN terminal differentiation in the posterior-most abdominal segments

Veverytsa and Allan (2012) have suggested that the ecdysone-induced nuclear hormone receptor signalling cascade triggers the terminal differentiation of the late CCAP-ENs in pupae (Veverytsa and Allan, 2012); misexpression by heat shock of the orphan nuclear receptor Ftz-f1, a central player in the ecdysone-induced nuclear hormone receptor cascade (Broadus et al., 1999; Woodard et al., 1994), in larvae 1, resulted in precocious differentiation of CCAP-ENs in segments A5–7 soon after the heat shock.

**Fig. 6.**
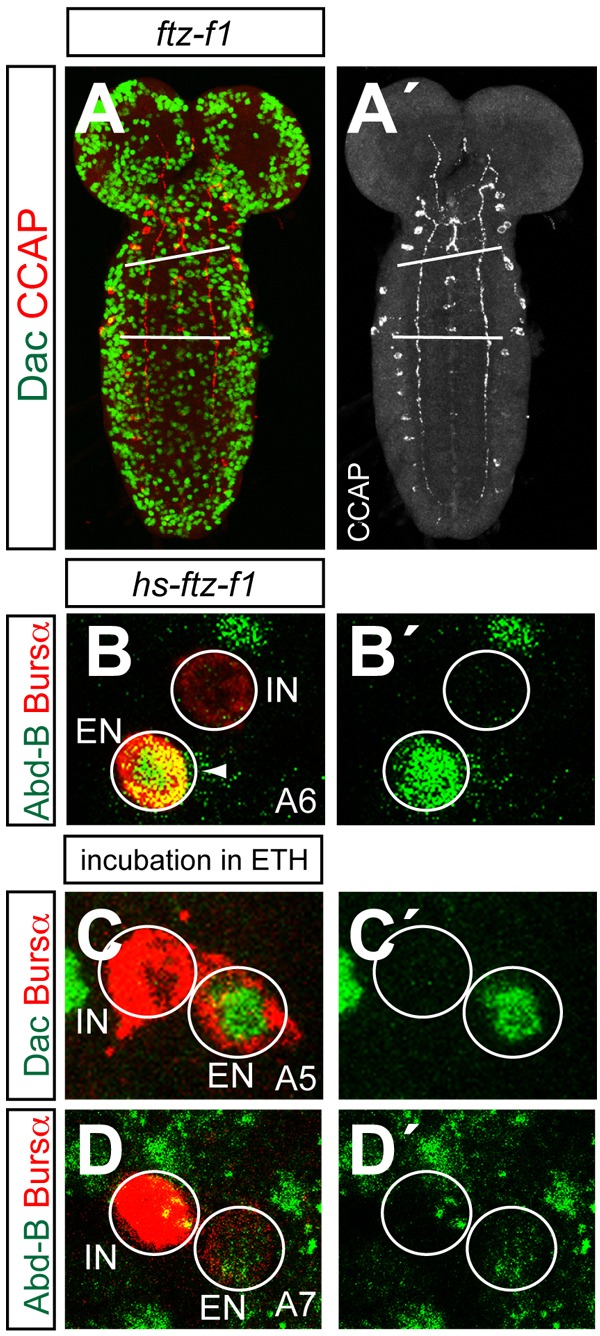
**Effects of the ecdysone pathway on the expression of CCAP and Bursα.** (A,A′) Expression of Dac (green) and CCAP (red) in *ftz-f1^Df(3L)BSC844^* first instar larvae. The red channel is shown separately in grey (A′). CCAP expression is weaker. White bars indicate subesophagus/thorax/abdomen boundaries. (B) Expression of Abd-B (green) and Bursα (red) in the A6 segment of an *hs-ftz-f1* early second instar larva heat shocked in late L1. An extra CCAP neuron expresses Abd-B (arrowhead). (C-D′) Expression of Bursα (red), Dac (green in C,C′) and Abd-B (green in D,D′) in the indicated abdominal segments of early larva 2 ganglia, incubated in an ETH solution and stained 4 h later.

This observation supports the idea that, in normal development, the ecdysone pulse in late larvae/early pupae triggers the terminal differentiation of CCAP neurons (Richards, 1981). If so, activation of Ftz-f1 may counteract repression of CCAP by Abd-B. We stained Bursα in larvae carrying different loss-of-function alleles of *ftz-f1* (*ftz-f1^ex19^*, *ftz-f1^ex7^* and *ftz-f1^Df(3L)BSC844^*) and observed that expression, although not absent, was very weak, mostly in abdominal segments (Fig. 6A-A′ and data not shown). We also reproduced the result obtained by the misexpression of *ftz-f1* (*hs-ftz-f1* larva aged from 12 to 36 h. after hatching were heat shocked at 37°C during 1 h. and dissected and stained 4 h. later), and occasionally observed extra CCAP-ENs, a phenotype not observed in wild-type larvae (Fig. 6B-B′).

The release of ecdysis-triggering hormone (ETH) by the Inka cells on tracheal tubes activates the ecdysis behavioural sequence via direct and sequential activation of several neuropeptides in the CNS (Ewer et al., 1997; Kim et al., 2006). In *Manduca sexta* injection of ETH promotes the release of CCAP from the 27/704 neurons, which are considered the homologs of the CCAP neurons in the moth (Ewer et al., 1997). We assessed the ability of ETH to overcome repression of the terminal differentiation of CCAP-ENs by Abd-B. To that end we incubated CNS of late second instar larvae for 2 h in a 2 mM solution of ETH in PBS, and 4 h. later stained for Bursα expression. We observed extra CCAP-ENs in several abdominal segments, a phenotype not observed in control larvae incubated in PBS. These ENs, which can be identified by the expression of Dac, express low levels of Abd-B (Fig. 6C-D′).

Ecdysone expression peaks several times during development (Richards, 1981). Three of these peaks, those occurring in the embryo and in the early and mid pupa, are distinguished by their intensity. Our results suggest that the peak occurring in early pupae counteracts the repression of CCAP neuropeptide by Abd-B. Thus, when we mimicked this peak in early larval development, we overcame prematurely the repression of neuropeptide production by Abd-B.

### Homothorax/Meis1 is not required for Ubx-mediated CCAP-EN specification

HOX gene products bind DNA in conjunction with DNA-binding cofactors, which increase their binding specificity (Mann et al., 2009). In *Drosophila*, the best-characterized cofactors are the TALE homeodomain proteins Extradenticle/Pbx (Exd/Pbx) and Homothorax/Meis1 (Hth/Meis1). *In vitro* studies show that most HOX proteins require Exd/Hth to bind effectively to many of their targets (Chan et al., 1994; van Dijk and Murre, 1994). Unlike most HOX genes, Abd-B does not appear to need to interact with Exd and Hth and, their presence even seems to interfere with the direct activation of *ems* by Abd-B in the posterior spiracles (Jones and McGinnis, 1993; Rivas et al., 2013; Sambrani et al., 2013). Abd-B represses *hth* transcription in the ectoderm, although in the larval CNS this repression is mostly directed by miRNAs-iab4/8 (Garaulet et al., 2014). We wherefore examined the requirement for Exd/Hth for specification of the CCAP neurons.

As *hth* loss of function resembles the complete absence of *exd,* and *hth* is not maternally provided (Peifer and Wieschaus, 1990; Rauskolb et al., 1993; Rieckhof et al., 1997), we assessed the requirement for Hth by staining CCAP and Bursα expression in loss-of-function mutants (*hth^5E04^*/*hth^6158^*) and in misexpression experiments (*elav-Gal4 UAS-hth*). Since we did not observe any phenotype (supplementary material Fig. S7A-B) we conclude that Hth is not required for specification of the CCAP-ENs.

## DISCUSSION

**Fig. 7.**
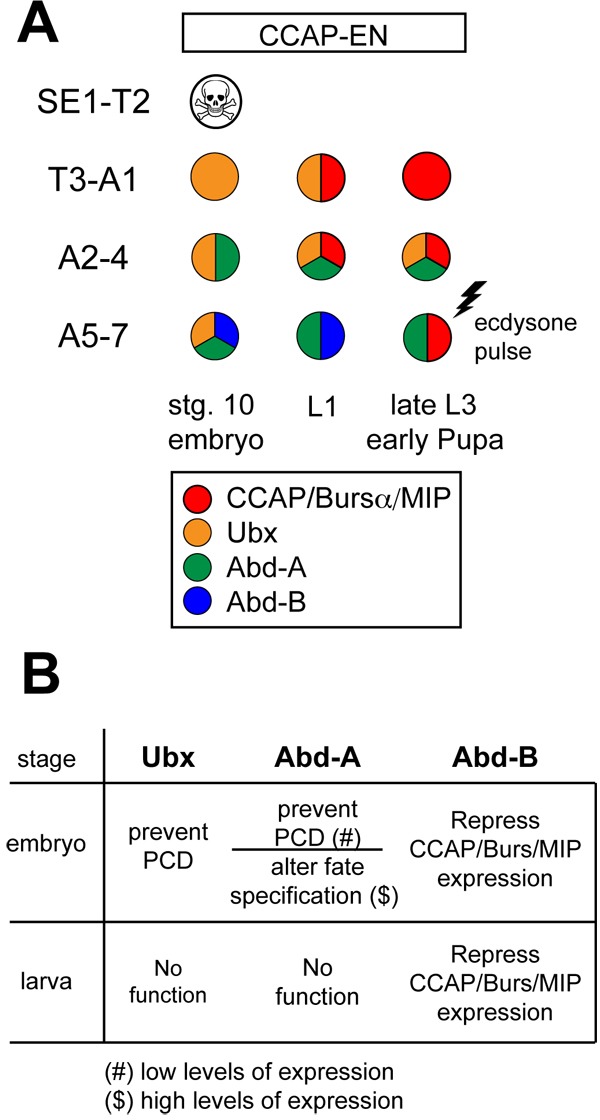
**Functions of the BX-C genes in patterning the CCAP-ENs.** (A) Summary scheme of CCAP/Bursα and HOX gene expression in the CCAP-ENs of stage 10 embryos, larvae 1 and late larvae3/early pupae. The different levels of expression of HOX genes in different segments are not represented. (B) Table summarizing the roles of Ubx, Abd-A and Abd-B in patterning CCAP-ENs in embryo and larva.

The results presented here permit several conclusions about the role of the BX-C genes in patterning the CCAP neurons: first, the only function of Ubx and Abd-A seems to be to prevent apoptosis of the CCAP-ENs in segments T3–A7 (Fig. 7). Once this early stage is passed, these gene products do not seem to be required for the maintenance of these neurons. Secondly, unlike what happens with Ubx, the level of Abd-A expression is determinant, since a high level of expression changes the fate of both CCAP-INs and -ENs. Third, unlike Ubx and Abd-A, which define the spatial pattern of CCAP-ENs, Abd-B controls the temporal onset of neuropeptide expression, such that the latter is not activated till either Abd-B is removed or its function is counteracted by activation of the ecdysone pathway. Further experiments are required to establish how this happens at the molecular level.

Our results do not tell us whether expression of Abd-B in the ENs of segments A5–7 is sufficient to prevent apoptosis of these neurons, since to visualize them *Abd-B* has to be removed, and in this situation they survive but express Ubx (data not shown). Strikingly, Abd-B prevents apoptosis of the MP1 and dMP2 pioneer neurons in the posterior segments in late embryonic stages (Miguel-Aliaga and Thor, 2004), but promotes apoptosis of Va neurons in the posterior-most segments (Suska et al., 2011). It will be interesting to know how the cellular context controls these opposite outcomes and whether the *Drosophila* pro-apoptotic genes *hid*, *reaper* and *grim* are direct targets of the BX-C genes, as has been shown for the HOX genes *Dfd* and *reaper* (Lohmann et al., 2002).

### The importance of temporal control in the terminal differentiation of neurons during development

Remodelling of the CNS to adapt to new environmental conditions is quite common in insects (Levine et al., 1995; Yu and Schuldiner, 2014). The most striking cases are observed in holometabolous insects, in which metamorphosis allows the larval CNS to be re-specified for adult functions that include the control of flight, walking and reproductive behaviour (Tissot and Stocker, 2000). Among the plethora of changes observed at metamorphosis is the terminal differentiation of neurons, which, until that stage, have remained dormant since the time they arose in embryonic or larval neurogenesis. Such is the case for the CCAP-ENs in segments A5–7: they are generated in the embryo but neuropeptide production only begins in the pupa (Veverytsa and Allan, 2012). Similar delays in the onset of neuropeptide expression and morphological differentiation have been observed in other neurons (Consoulas et al., 2002; Schneider et al., 1993), but the underlying mechanisms are poorly understood.

Due to the diversity of functions performed by the different segments of the adult organism, it seems likely that the HOX genes play a crucial role in translating the global temporal clues that promote metamorphosis into segment-specific transformations. This is the case for *Dfd*, which is specifically required for the reorganization of the CNS in the head during metamorphosis (Restifo and Merrill, 1994).

It has been suggested that the delayed terminal differentiation of this set of CCAP neurons is due, either to a distinctive connectivity of these neurons that is specifically required for pupal ecdysis, or a requirement for a (high) level of CCAP secretion for pupal ecdysis that would be detrimental for larval ecdysis (Veverytsa and Allan, 2012). Unlike other neuropeptide receptors, the CCAP receptor requires a relatively high concentration of ligand to be activated (Park et al., 2002). Although this finding supports the latter model, it does not exclude the first. We did not observe any effect of overexpressing CCAP or Burs (*CCAP-Gal4 UAS-CCAP* or *CCAP-Gal4 UAS-Burs*; data not shown) on the behavioural changes preceding ecdysis to the third larval instar, which supports a specific role of the late ENs in driving pupal ecdysis.

*ftz-f1* is widely expressed in the nervous system and has been reported to be an important downstream component of the ecdysone cascade that drives the terminal differentiation or re-modelling of many neurons (Ohno and Petkovich, 1993; Ruaud et al., 2010; Yamada et al., 2000). For instance, it has been implicated in remodelling the morphology of the neurons of the mushroom body (Boulanger et al., 2011). In the same way, misexpression of *ftz-f1* in early second instar larvae is sufficient to initiate expression of CCAP and Bursα in the CCAP-EN of segments A5–7, a process that normally happens at the end of larval life (this work; see also Veverytsa and Allan, 2012). However, it is not known how this is achieved.

In this report we have shown that, during normal development, Abd-B represses the expression of CCAP/Bursα in the CCAP-EN of the posterior-most abdominal segments, and when misexpressed is able to repress neuropeptide expression in all CCAP neurons. Further, *Abd-B* knock-down in larvae results in precocious onset of CCAP/Bursα production. When *ftz-f1* was misexpressed, we observed, in abdominal segments, extra CCAPs that expressed Abd-B, which suggests that Ftz-f1 counteracts repression by Abd-B of the expression of neuropeptides. We obtained the same result incubating second instar larval ganglia in a solution containing ETH. Thus, the expression of CCAP/Burs in these neurons requires either removal of Abd-B or activation of the ecdysone cascade to counteract the repressor effect of Abd-B on neuropeptide expression. These findings provide a segment-specific mechanism that explains the differences in behaviour of CCAP-ENs.

Abd-B seems to play a similar role in the ABLK neurons of the A8 segment, repressing the expression of the neuropeptide Lk. These neurons are present in segments A1–7. In *Abd-B* mutants an extra ABLK per hemisegment appears in A8 and the same phenotype is observed when *Abd-B* is knocked down from first instar larva, at a time when embryonic neurogenesis is complete, suggesting that the role of Abd-B is to prevent the expression of Lk is this segment, but that it is not required for production of this neuron. Unlike the expression of CCAP/Burs in the CCAP-ENs of segments A5–7, the repression of Lk in A8 segment is permanent (Estacio-Gomez et al., 2013).

The huge diversity of effects that HOX genes of the BX-C have on patterning of the CNS is noteworthy (reviewed in Estacio-Gómez and Díaz-Benjumea, 2014). In many cases the same gene product drives opposing effects in different cells, which highlights the importance of the cellular context and the fact that, in contrast to the role they have in imaginal discs, where they act as selector genes (reviewed in Mann and Morata, 2000), in the CNS, HOX genes are part of the complex combinatorial codes that define neuronal fates. This, on the one hand, highlights the need to identify more cofactors to help us understand how these genes act (reviewed in Mann et al., 2009), and on the other, emphasises the temporal component of their actions, since in some cases they are required at a specific stage of development – this is the case for Ubx/Abd-A in the specification but not the maintenance of the CCAP-EN fate, or the pulse of Abd-A expression ending proliferation of abdominal neuroblasts (Maurange et al., 2008) – while in other cases they are required permanently for a period of time, as in Abd-B repression of neuropeptide expression during larval development.

## MATERIALS AND METHODS

### Fly strains

The fly stocks used were as follows: *lab^1^*, *lab^4^*, *pb^10^*, *Dfd^10^*, *Scr^4^*, *Antp^14^*, *Antp^25^*, *Ubx^6.28^*, *Ubx^9.22^*, *Ubx^MX6^*, *abd-A^M1^*, *abd-A^P24^*, *abd-A-lacZ*, *Abd-B^M8^*, *Abd-B^M5^* (this allele removes the m isoform), *Abd-B^M1^*, *Abd-B^D16^* (provided by F. Karch, Université de Genéve, Switzerland), *Abd-B^D18^* (these three last alleles remove both *Abd-B* isoforms), *Abd-B^Df(3R)C4^* (this deficiency remove *Abd-B* and the non-coding RNAs; provided by F. Karch), *Df(3R)109* (remove *Ubx* and *abd-A*), *Df(3L)H99* (this deficiency removes *hid, rpr* and *grim*)*, hth^5E04^, hth^Df(3R)Exel6158^* (this deficiency removes *hth*), *dimm^rev7^* (provided by R. Hewes, University of Oklahoma, USA), *ftz-f1^ex7^* and *ftz-f1^ex19^* (provided by J-M. Dura, Institut de Gènètique Humaine, Montpellier, France) *ftz-f1^Df(3L)BSC844^* (this deficiency removes *ftz-f1*) and *hs-ftz-f1* (provided by C. Woodard, Mount Holyoke College, USA).

Gal4/Gal80 lines: *worn-Gal4, elav-Gal4^C155^*, *CCAP-Gal4* (provided by Ch. Wegener, Universität Würzburg, Germany), *tub-Gal80^ts^*.

UAS lines: *UAS-GFP*, *UAS-lab, UAS-pb, UAS-Dfd^w4^, UAS-Scr, UAS-Antp*, *UAS-UAS-Ubx^IAI^*, *UAS-abd-A^20-1-1^*, *UAS-abd-A^20-10-1^*, *UAS-Abd-B^M2SG19^*, *UAS-hth* (long isoform; provided by N. Azpiazu, CBMSO, Madrid, Spain), *UAS-dsUbx*, *UAS-Abd-B-RNAi* (Vienna Drosophila Resource Centre) and *UAS-dicer2*. The Bloomington Drosophila Stock Centre at Indiana University (USA), unless otherwise indicated, provided these fly stocks.

### Immunohistochemistry and confocal imaging

Primary antibodies used were: mouse anti-GFP (1:200; Roche, #11814460), rabbit anti-GFP (1:200; Invitrogen, #A6455); rabbit anti-Lk (1:100) provided by D. Nässel (Stockholm University, Sweden); rat anti-CCAP (1:100) (Moris-Sanz et al., 2014); rabbit anti-Bursα (1:250) provided by R. White (National Institute of Mental Health, Bethesda, USA); rabbit anti-Mip (1:1000) and rabbit anti-Crz (1:1000) provided by J. Veenstra (INCI, Bordeaux, France); guinea pig anti-Dimm (1:100) provided by P. Taghert (Washington University, St. Louis, USA); guinea pig anti-Hb (1:200) provided by I. Miguel-Aliaga (Imperial College, London, UK); rat anti-Abd-A (1:500) provided by J. Casanova (IRB, Barcelona, Spain); rabbit anti-Abd-A (1:50) provided by M. Capovilla (Agrobiotech Institute, Sophia Antipolis, France); rat anti-Ems (1:10) provided by U. Walldorf (University of Saarland, Homburg/Saar, Germany); mouse anti-Ubx (1:20; DSHB, #FP3-38); mouse anti-Abd-B (1:50; DSHB, #1A2E9); mouse anti-Dac (1:50; DSHB, #mAbdac2-3) and chicken anti-β-galactosidase (1:400; Abcam, #ab9361).

Immunostaining was performed according to (Benito-Sipos et al., 2010), and confocal image stacks were collected using a Zeiss LSM710 or LSM510 confocal microscope.

### ETH incubation

Late second instar larva were dissected in PBS and incubated in 0.1 mg/ml ETH peptide (DDSSPGFFLKITKNVPRL) solution in PBS at 25°C for 2 h. After several washes, ganglia were kept in PBS for 4 h. and then fixed and stained.

## Supplementary Material

Supplementary information
