# Supplementary material for: The study of the Bithorax-complex genes in patterning CCAP neurons reveals a temporal control of neuronal differentiation by Abd-B
Source: Biol Open. 2015 Aug 14;4(9):1132–42. doi: 10.1242/bio.012872 (PMC4582124; doi:10.1242/bio.012872)
Supplement: Supplementary information [file supp_4_9_1132__index.html]

The study of the Bithorax-complex genes in patterning CCAP neurons reveals a temporal control of neuronal differentiation by Abd-B — Supplementary information 

# The study of the Bithorax-complex genes in patterning CCAP neurons reveals a temporal control of neuronal differentiation by Abd-B

## BIO012872 Supplementary information

**Files in this Data Supplement:**

- Supplementary information
