# Supplementary material for: The study of the Bithorax-complex genes in patterning CCAP neurons reveals a temporal control of neuronal differentiation by Abd-B
Source: Biol Open. 2015 Aug 14;4(9):1132–42. doi: 10.1242/bio.012872 (PMC4582124; doi:10.1242/bio.012872)
Supplement: Supplementary information [file supp_bio.012872_BIO012872supp.pdf]

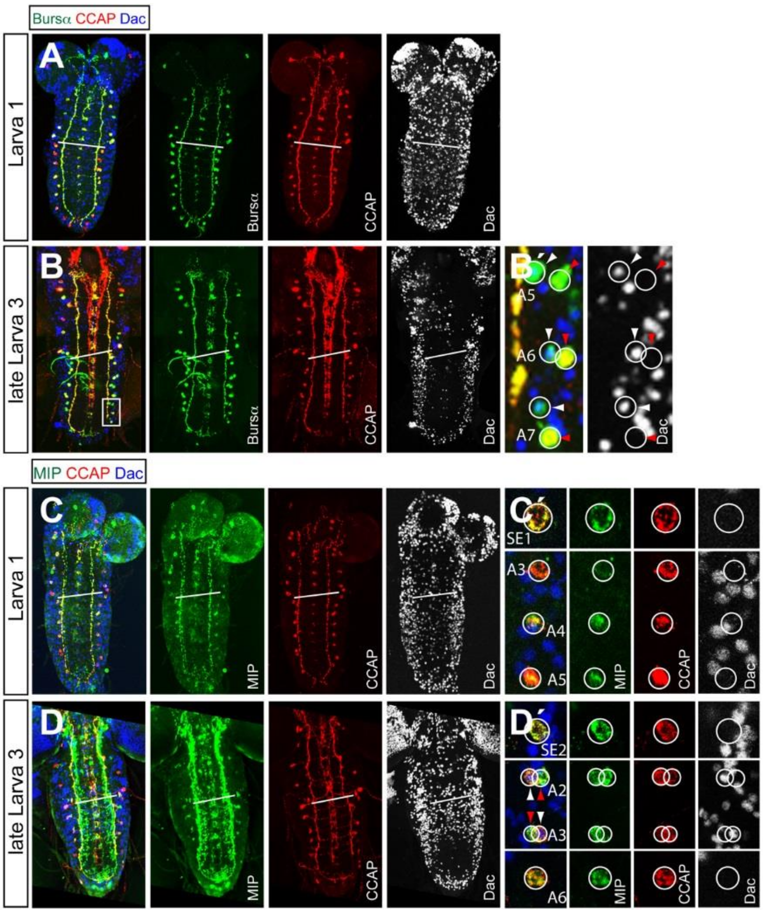

Figure S1. Pattern of expression of CCAP, Bursα and MIP. (A) Staining for Bursα (green), CCAP (red) and Dac (blue) in wild-type first (A) and third (B-B′) instar larvae. Merged and separate channels are shown. (B′) Magnified view of hemisegments A5–7 (area selected in B). (C-D) Staining for MIP (green), CCAP (red) and Dac (blue) in wild-type first (C) and third (D) instar larvae. Merged and separate channels are shown. (C′ and D′) Closer views of the indicated hemisegments of first (C′) and third (D′) instar larvae. White and red arrowheads indicate CCAP-ENs and INs respectively. White bar indicates thorax/abdomen separation.

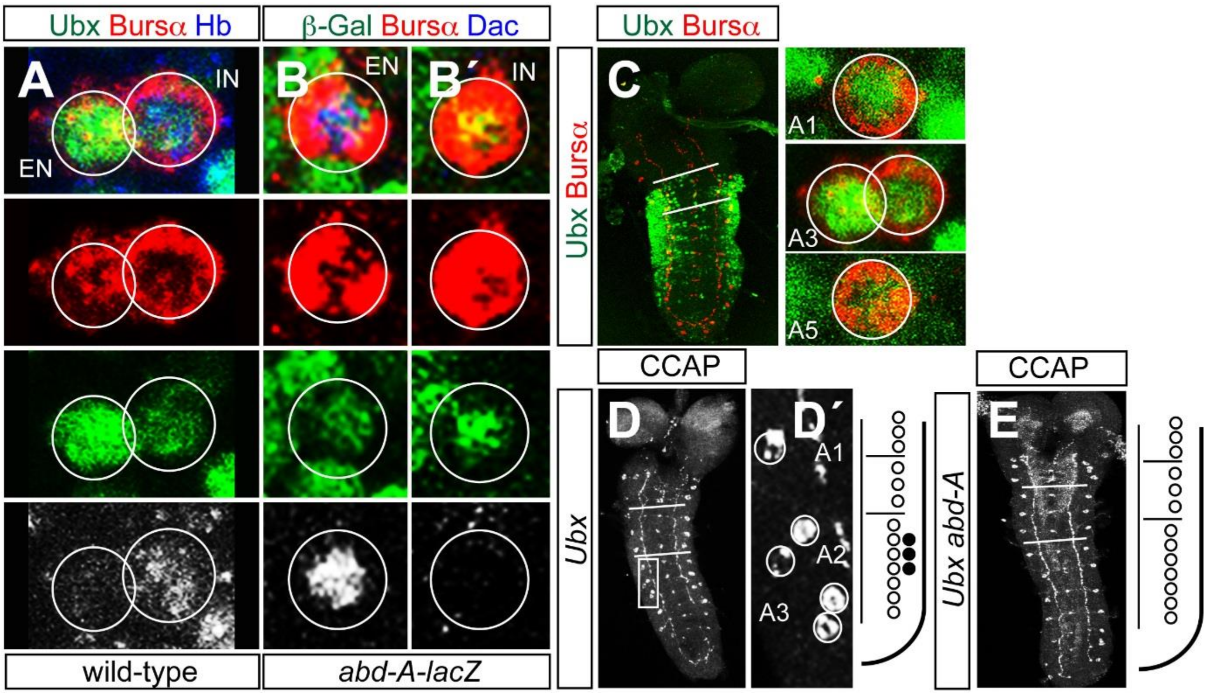

Figure S2. (A-B′) Unmerged channels of Figures 2A, B and B′. (C) Expression of Ubx (green) and Bursα (red) in wild-type larvae 1. (D-E) Expression of CCAP in *Ubx*$^{6.28}$ (D) and *Ubx*^{MX6} *abd-A*^{M1} (E) larvae 1. The area selected in (D) is magnified in (D´) and corresponds to segments A1-3. CCAP neurons (circles) are indicated. CCAP-EN is missing in T3–A1 in (D) and in all segments in (E). White bars indicate subesophagus/thorax/abdomen boundaries.

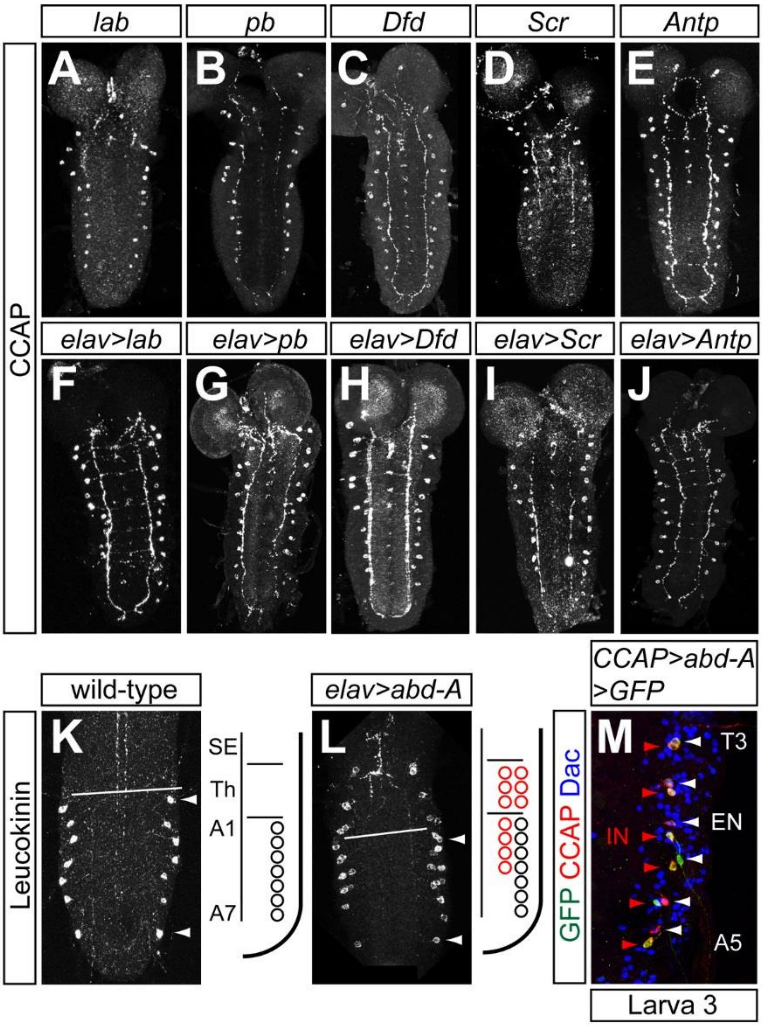

Figure S3. (A-J) Expression of CCAP in *lab*^{1}/*lab*^{4} (A), *pb*^{10} (B), *Dfd*^{10} (C), *Scr*^{4} (D), *Antp*^{14}/*Antp*^{25} (E), *elav-Gal4 UAS-lab* (F), *elav-Gal4 UAS-pb* (G), *elav-Gal4 UAS-Dfd*^{w4} (H), *elav-Gal4 UAS-Scr* (I) and *elav-Gal4 UAS-Antp* (J) ganglia of larvae 1. (K-L) Expression of Lk in wild-type (K) and *elav-Gal4 UAS-abd-A* (L) larva 1 ganglia. Diagrams depicting the phenotypes are shown on the right of (K-L). Black circles: ABLK neurons; red circles: ectopic ABLKs. White bars indicate thorax/abdomen separation. (M) Expression of CCAP (red) and Dac (blue) in *CCAP-Gal4 UAS-abd-A UAS-GFP* (green) third instar larva. T3–A5 segments are shown. White and red arrowheads indicate CCAP-EN and -IN respectively.

Biology Open • Supplementary information

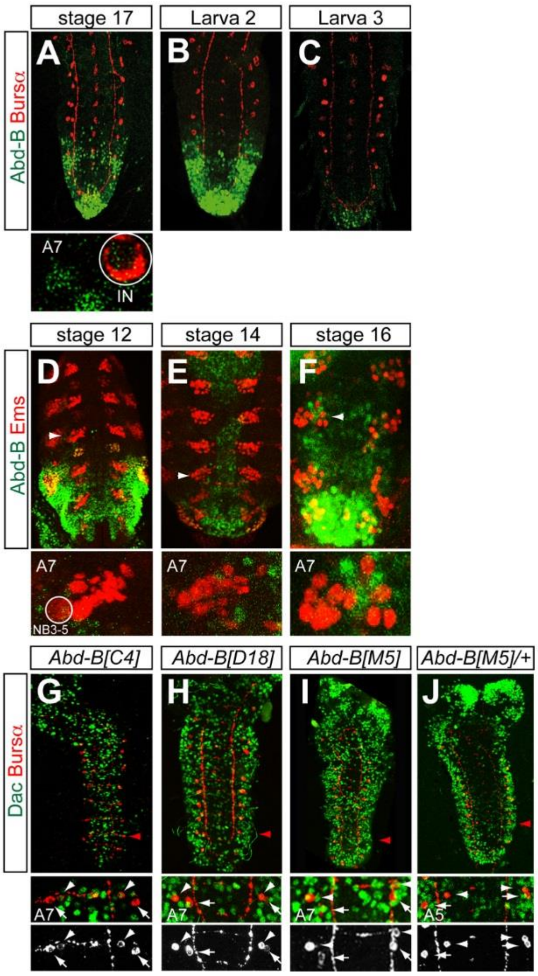

Figure S4. Abd-B expression in the NB3-5 cluster. (A-C) Expression of Abd-B (green) and Bursα (red) in wild-type ganglia of a stage 17 embryo (A), larva 2 (B) and larva 3 (C). Abd-B expression is never detected in CCAP-INs or CCAP-ENs. (D-F) Expression of Abd-B (green) and Ems (red) in ganglia of embryos of stage 12 (D), 14 (E) and 16 (F). High magnifications of the NB3-5 cluster are shown at the bottom. Expression of Abd-B is not detectible in the NB3-5 cluster. (G-J) Expression of Dac (green) and Bursα (red) in *Abd-B*$^{Df(3R)C4}$ (G), *Abd-B*^{D18} (H), *Abd-B*^{M5} (I) and *Abd-B*^{M5}/+ (J) ganglia of larva 1. In (G-J) magnified views of A7 (G-I) or A5 (J) segments (red arrowhead) are shown at the bottom of each figure; white arrows: CCAP-INs, white arrowheads: CCAP-ENs. The red channel is shown separately in white.

Biology Open • Supplementary information

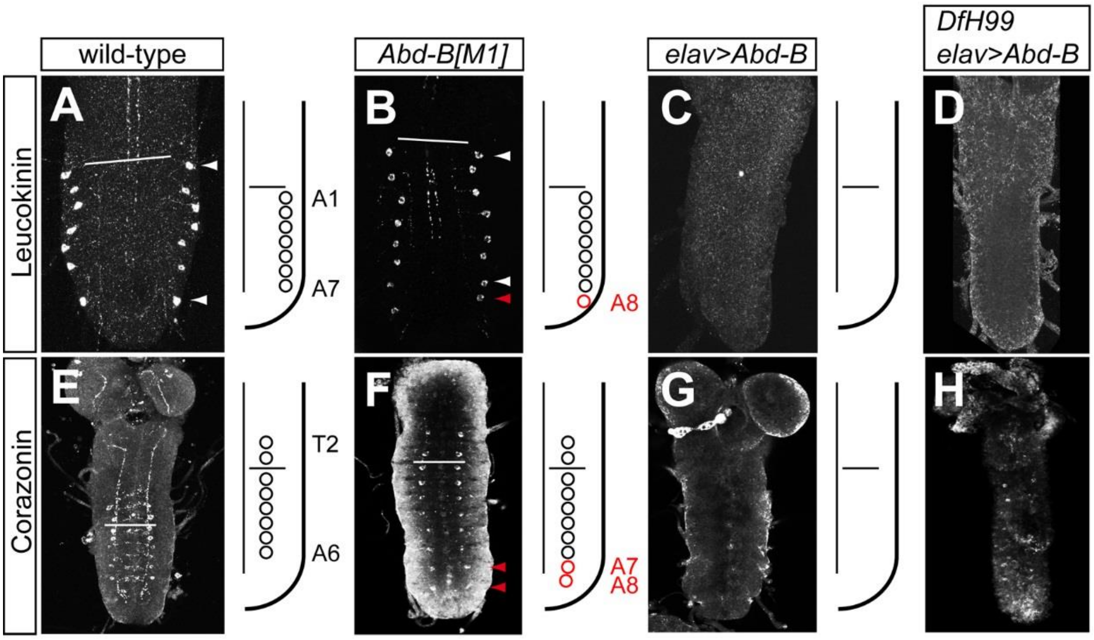

Figure S5. Abd-B controls the expression of Leucokinin and Corazonin neuropeptides. (A-L) Staining for the expression of the neuropeptides Lk (A-D) and Crz (E-H) in wild-type (A and E), *Abd-B^{M1}* (B and F), *elav-Gal4 UAS-UAS-Abd-B^{M2}* (C and G) and *Df(3L)H99 elav-Gal4 UAS-Abd-B^{M2}* (D and H). Diagrams showing the phenotypes are on the right of each figure. The expression of Lk and Crz in the brain is not shown. White arrowheads in (A-B) indicate A1 and A7 segments. Red arrowheads in (B and F) and red circles in the diagrams indicate extra neuropeptide-expressing neurons. White bars indicate thorax/abdomen separation.

Biology Open • Supplementary information

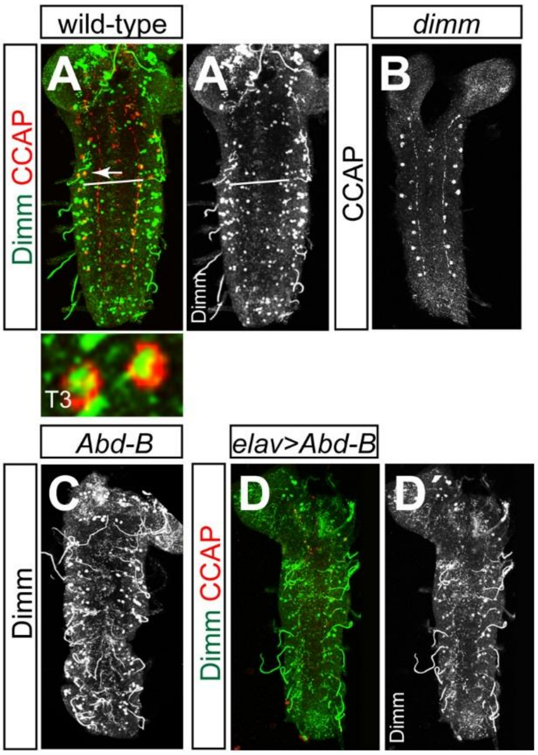

Figure S6. *Abd-B* misexpression does not alter the expression of Dimm

(A-A′) Expression of Dimm (green) and CCAP (red) in wild-type ganglia of larvae 1. White bars indicate thorax/abdomen separation. A high magnification of the T3 hemisegment (arrow) is shown at the bottom. The green channel is shown separately in (A′). Both CCAP neurons express Dimm. (B) CCAP expression in $dimm^{rev7}$. (C) Dimm expression in $Abd\text{-}B^{M1}$ ganglion of larva 1. (D-D′) Expression of Dimm (green) and CCAP (red) in *elav-Gal4 UAS-*$Abd\text{-}B^{M2}$ ganglion of larva 1. The green channel is shown separately in (D′). Dimm expression is not affected by misexpression of *Abd-B*.

Biology Open • Supplementary information

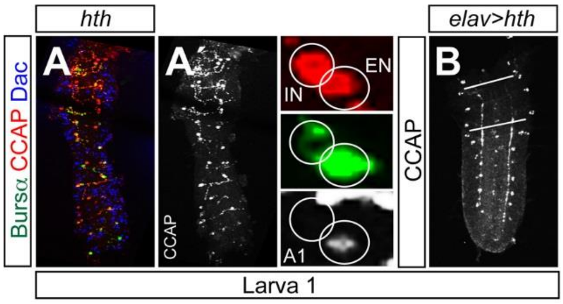

Figure S7. Hth does not alter the expression of CCAP/Bursα

(A-A′) Expression of Bursα (green), CCAP (red) and Dac (blue) in a *hth* $^{Df(3R)Exel6158}$/*hth*^{5E04} ganglion of larva 1. The red channel is shown in (A′). Separate channels of hemisegment A1 are shown on the right. (B) Expression of CCAP in *elav-Gal4 UAS-hth* (long isoform) ganglion of larva 1. CCAP expression was not affected in these experime nts. White bars indicate subesophagus/thorax/abdomen separation.

Biology Open • Supplementary information

Supplementary Material Table S1

		Subesphagus			Thorax			Abdomen		
		n	AV	SD	n	AV	SD	n	AV	SD
	Wild-type	70	2,8	0,5	73	3,9	0,2	74	7,7	1,64
	Wild-type (#)	20	3,0	0,0	20	4,0	0,0	20	10,4	1,9
	Wild-type LIII	19	2,7	0,5	19	3,6	0,6	19	7,9	1,2
	Wild-type (#) LIII	20	2,8	0,3	20	3,8	0,4	20	12,1	1,4
Hox genes	*lab*^{1}/*lab*^{4}	20	2,8	0,5	20	3,8	0,5	20	6,8†	0,7
	pb^{10}	12	2,6	0,5	14	3,9	0,3	14	6,5†	0,9
	Dfd^{10}	20	2,8	0,6	21	3,9	0,2	20	8,5	1,1
	Scr^{4}	10	3,1	0,3	10	3,7	0,5	10	6,7†	0,7
	Antp^{14}/*Antp*^{25}	26	3,1(†)	0,5	26	4,1	0,7	10	7,4	0,7
	Ubx$^{6.28}$	15	3,0†	0,0	15	3,2*	0,4	15	6,9	1,6
	Ubx$^{6.28}$ (#)	20	3,2	0,5	20	3,0*	0,0	20	9,45	1,3
	elav^{C155}*-Gal4 UAS-dicer2 tub-Gal80*^{ts} *UAS-dsUbx Df(3R)109* LIII (#)	20	3,3	0,5	20	4	0,0	20	13,2	0,9
	Df(3L)H99	9	5,4†	1,8	10	4,2	1,2	8	6,5	1,5
	Df(3L)H99 (#)	9	4,5*	0,5	11	5,8*	0,6	11	9,9	1,8
	Df(3L)H99 Ubx$^{6.28}$ (#)	10	4,9*	1,4	10	5,1*	1,0	10	10,2	1,5
	Ubx^{MX6} *abd-A*^{M1}	21	2,9	0,4	23	2,8*	0,5	21	6,9†	0,6
	Ubx^{MX6} *abd-A*^{M1} (#)	11	2,9	0,3	14	3,0*	0,0	14	7,0*	0,0
	Df(3L)H99 Ubx^{MX6} *abd-A*^{M1} (#)	6	4,2*	0,9	6	5,3*	0,5	6	9,8	0,6
	abd-A^{M1}	25	2,9	0,4	33	3,5	1,1	32	6,9†	0,5
	abd-A^{M1} (#)	14	2,7	0,4	14	3,6	0,6	14	11,1	0,2
	Abd-B^{M1}	19	3,0	0,3	19	4,0	0,0	19	10,5*	1,7
	Abd-B^{M1} (#)	19	3,0	0,3	19	4,0	0,0	19	16*	2,3
	Abd-B^{M5} (#)	20	3,0	0,0	20	3,9	2,3	20	14,4*	0,8
	Abd-B^{M5}/+ (#)	20	2,8	0,3	20	3,9	0,2	20	11,9	0,5
	Abd-B$^{Df(3R)C4}$ (#)	12	2,7	0,4	12	4,0	0,0	12	13,4*	1,3
	Abd-B^{D16} (#)	15	2,8	0,3	15	4,0	0,0	15	13,6*	0,6
	Abd-B^{D18} (#)	14	3,0	0,0	16	3,7	0,2	16	13,5*	0,7
	elav-Gal4^{C155} *UAS-Abd-B-RNAi*^{12024}	18	3,1	0,4	28	4,0	0,0	36	14,6*	1,4
	worn-Gal4 UAS-lab	49	3,0†	0,2	49	3,8	0,4	43	7,1	1,5
	worn-Gal4 UAS-pb	15	2,8	0,5	17	3,8	0,3	17	8,0	1,0
	worn-Gal4 UAS-Dfd^{W4}	15	2,9	0,2	18	3,7	0,4	19	7,1	0,9

	worn-Gal4 UAS-Scr	15	2,7	0,4	15	3,9	0,2	16	8,1	1,2
	worn-Gal4 UAS-Antp	15	3,0	0,6	14	3,9	0,5	18	7,1	2,0
	worn-Gal4 UAS-Ubx^{IAI}	33	2,7	0,8	33	3,5	1,0	18	7,8	1,6
	worn-Gal4 UAS-abd-A$^{20\text{-}10\text{-}1}$	15	3,3	1,1	15	4,0	0,2	15	7,0	1,2
	worn-Gal4 UAS-Abd-B^{M2SG19}	13	2,9	0,3	13	3,8	1,3	13	8,6	2,4
	elav-Gal4^{C155} *UAS-lab*	12	2,8	0,3	12	4,0	0,0	12	8,2	1,3
	elav-Gal4^{C155} *UAS-pb*	35	3,0†	0,0	36	4,0	0,3	38	7,4	3,0
	elav-Gal4^{C155} *UAS-Dfd*^{W4}	18	3,0†	0,0	18	3,9	0,2	19	7,4	1,0
	elav-Gal4^{C155} *UAS-Scr*	55	2,7	0,5	47	3,7	0,4	17	7,0	0,6
	elav-Gal4^{C155} *UAS-Antp*	15	2,9	0,2	16	3,8	0,2	15	7,0	0,7
	elav-Gal4^{C155} *UAS-Ubx*^{IAI}	17	3,5*	0,5	18	5,5*	1,0	20	6,9†	0,7
	elav-Gal4^{C155} *UAS-Ubx*^{IAI} (#)	26	3,9*	0,9	26	4,7*	0,7	26	9,5	1,2
	elav-Gal4^{C155} *UAS-abd-A*$^{20\text{-}10\text{-}1}$	48	0,08*	0,28	50	0,0*	0,0	50	0,04*	0,2
	elav-Gal4^{C155} *UAS-abd-A*$^{20\text{-}10\text{-}1}$ (#)	20	0,3*	0,7	20	0,2*	0,5	20	0,1*	0,5
	elav-Gal4^{C155} *UAS-abd-A*^{II}	19	0,3*	0,7	19	0,1*	0,5	19	0,1*	0,5
	Df(3L)H99 elav-Gal4 UAS-abd-A	12	0,6	0,7	12	0,7	0,9	12	0,6	0,8
	elav-Gal4^{C155} *UAS-Abd-B*^{M2SG19}	16	0,0*	0,0	16	0,0*	0,0	16	0,0*	0,0
	Df(3L)H99 elav-Gal4 UAS-Abd-B^{M2SG19}	10	0,2*	0,4	10	0,6*	1,1	10	1,0*	2,1
	CCAP-Gal4 UAS-abd-A$^{20\text{-}10\text{-}1}$ LIII	9	3	0,2	11	3,7	0,5	11	8,6	2,5
	CCAP-Gal4 UAS-Abd-B^{M2SG19} LIII	10	0,3*	0,7	10	0,5*	0,7	10	1,1*	0,9
Other genes	*dimm*^{rev7}	18	2,9	0,3	18	4,0	0,0	18	6,8†	0,7
	hth$^{Df(3R)Exel6158}$*/hth*^{5E04}	15	2,3	1,0	19	3,2	0,8	21	7,2	3,5
	elav-Gal4^{C155} *UAS-hth*	16	2,9	0,6	16	4,0	0,0	16	7,2	1,0
	ftz-f1$^{Df(3L)BSC844}$	10	1,7*	1,6	10	1,3*	1,4	10	0,5*	1,1
	ftz-f1^{ex19}	16	3,8	0,3	16	3,4	0,7	16	5	1,1
	ftz-f1^{ex7}	19	3,0	0,4	20	4,0	0,0	20	6,7	1,1
	hs-ftz-f1 (#)	21	2,8	0,2	20	3,9	0,2	22	10,5	0,2
	Wild-type (ETH incubation) LII	15	2,8	0,7	15	3,8	0,4	15	12,4	1,2

n: number of scored hemiganglia. All data correspond to early first instar ganglia, unless otherwise indicated. LII: second instar larva, LIII: late third instar larva.
AV: average number of CCAP or Bursα (#)-expressing neurons per subesophagic, thoraxic and abdominal hemiganglia.
SD: standart desviation.
T students test was aplied; (†) significant differences, p-value< 0.01; (*) significant differences, p-value< 0.001.
